# Creating the humanitarian professional: moving from certification to advocacy and endorsement

**DOI:** 10.1186/2052-3211-7-S1-O11

**Published:** 2014-12-17

**Authors:** George Fenton, Chris Wright, Becky Turner

**Affiliations:** 1 Humanitarian Logistics Association/World Vision, Gloucester, United Kingdom; 2 International Association for Public Health Logisticians (IAPHL)/John Snow Inc., Addis Ababa, Ethiopia; 3 Humanitarian Logistics Association/Centers for Disease Control and Prevention, Port au Prince, Haiti

## Background

Health Logistics Association (HLA) is an association of logistics professionals committed to humanitarian logistics effectiveness by creating opportunities for dialogue and cooperative relationships with its members and partners to build a community of practice for advancing the humanitarian logistics profession through the promotion of cross organisational learning and collaboration. IAPHL is a professional association dedicated to improving public health supply chain management by promoting the professional development and recognition of those who work with health supplies.

## Method

Under the Enhanced Learning & Research for Humanitarian Assistance professionalization umbrella, the HLA has joined an inter-agency project to define a framework and system for recognising relevant humanitarian logistics experience and training. Initial research will be required to ensure that any framework is relevant, inclusive and aligned with other initiatives. The project will build on training program achievements in the humanitarian logistics (and emergency nutrition) sector and support the emerging suite of competence based qualifications.

## Results

Humanitarian assistance partners recognize the importance of effective humanitarian logisticians in the planning and delivery of aid. More humanitarian assistance professionals and academics see the need to hone specific competencies and skills for these programs so certifications build a portfolio of evidence. A key challenge is to ensure that individuals successfully completing these and other relevant qualifications gain the recognition they require from aid agencies. There is also a need to define a recognised career pathway within the sector with clear routes for progression. IAPHL is investigating professional certification requirements and seeks to learn from HLA’s experience.

## Discussion

The current system for recognizing knowledge and skills of humanitarian workers strongly favours international staff. Limited professional development funding for national staff is often wasted, as there is no accepted system to measure competencies or training effectiveness. A new system to capture, recognise and certify the skills, learning and development of aid workers is needed. It’s proposed that an internationally recognized Learning and Development Passport for humanitarian logisticians be development, and extended to include other related sectors such as public health logisticians.

## Lessons learned

Given HLA’s experience, the next steps for professionalizing the humanitarian logistics workforce are to stimulate greater advocacy, buy-in, recognition and endorsement for certification in the mainstream. HLA’s experience can be applied to other sectors like public health; IAPHL and PtD can build on and formalize the methods for professional certification and standards that have worked for HLA.

